# A Comparative QSAR Analysis, Molecular Docking and PLIF Studies of Some N-arylphenyl-2, 2-Dichloroacetamide Analogues as Anticancer Agents

**Published:** 2017

**Authors:** Masood Fereidoonnezhad, Zeinab Faghih, Ayyub Mojaddami, Zahra Rezaei, Amirhossein Sakhteman

**Affiliations:** a *Department of Medicinal Chemistry, School of Pharmacy, Ahvaz Jundishapur University of Medical Sciences, Ahvaz, Iran. *; b *Department of Medicinal Chemistry and Pharmaceutical Sciences Research Centre, School of Pharmacy, Shiraz University of Medical Sciences, Shiraz, Iran. *; c *Medicinal and Natural Products Chemistry Research Center, Shiraz University of Medical Sciences, Shiraz, Iran.*

**Keywords:** DCA, QSAR, *in silico* screening, Descriptor analysis, Docking, PLIF studies

## Abstract

Dichloroacetate (DCA) is a simple and small anticancer drug that arouses the activity of the enzyme pyruvate dehydrogenase (PDH) through inhibition of the enzyme pyruvate dehydrogenase kinases (PDK1-4). DCA can selectively promote mitochondria-regulated apoptosis, depolarizing the hyperpolarized inner mitochondrial membrane potential to normal levels, inhibit tumor growth and reduce proliferation by shifting the glucose metabolism in cancer cells from anaerobic to aerobic glycolysis. In this study, a series of DCA analogues were applied to quantitative structure–activity relationship (QSAR) analysis. A collection of chemometrics methods such as multiple linear regression (MLR), factor analysis–based multiple linear regression (FA-MLR), principal component regression (PCR), and partial least squared combined with genetic algorithm for variable selection (GA-PLS) were applied to make relations between structural characteristics and cytotoxic activities of a variety of DCA analogues. The best multiple linear regression equation was obtained from genetic algorithms partial least squares, which predict 90% of variances. Based on the resulted model, an *in silico*-screening study was also conducted and new potent lead compounds based on new structural patterns were designed. Molecular docking as well as protein ligand interaction fingerprints (PLIF) studies of these compounds were also investigated and encouraging results were acquired. There was a good correlation between QSAR and docking results.

## Introduction

There has been a great detonation in the number of potential molecular targets that can be investigated for cancer treatment. Some metabolic pathways that play a great role in tumor growth are being explored as novel targets for anticancer drug development (1, 2). Mitochondria are essential for the continuation of life in higher eukaryotic cells, including cancer cells. Several common characteristics of recognized tumor cells directly or indirectly depend on mitochondrial deregulation (3). Meanwhile, they control programmed cell death (apoptosis). Extensive investigation has been focused on the progression of strategies designed in order to selectively induce apoptosis in cancer cells (1, 4). Pyruvate dehydrogenase complex (PDC) is one of the major regulators of mitochondrial function. PDC is a complex of three enzymes that convert pyruvate into acetyl-CoA by pyruvate decarboxylation. PDC via production of reactive oxygen species (ROS) and followed by oxidative damage, can induce apoptosis. The activity of PDC is regulated by reversible phosphorylation of three serine residues on the E1α subunit. PDH kinases (PDK) phosphorylate these sites. There are four known isoforms of PDKs that are distributed in a different manner in the tissues. Their expressions are regulated by factors like hypoxia, starvation and employment of glucose and fatty acids in various tissues. It should be noted that the role of PDK (1–4) is inactivation of PDC (1, 5).

It was discovered that dichloroacetate (DCA) acts as a pyruvate dehydrogenase activator through stimulating PDC activity. DCA is a lactate-lowering drug, which has been in use for many years to treat various diseases such as lactic acidosis, inborn errors in mitochondrial function (6, 7). In 2007 it was discovered that the drug DCA induced the death of human lung, breast and brain cancer cells that were embedded into rats, while being non-toxic to healthy cells (8). DCA prevent cell growth of a large range of tumor cells like lung, breast, glioblastoma (8), endometrial (9), prostate (10), pediatric (11), pancreatic (12), cervical (13) and colorectal (14) cancer cells by promoting mitochondria-regulated apoptosis and decreasing proliferation. Nevertheless, it exerted no obvious toxicities on the normal cells.

Molecular modelling studies such as quantitative structure activity relationship (QSAR) and molecular docking have a great importance in the field of medicinal chemistry. There are different variable selection methods available for QSAR studies such as multiple linear regression (MLR), principal component or factor analysis (PCA ⁄ FA), genetic algorithm, and so on (15). Recently structure-based design of some PDK2 inhibitors from molecular docking studies has been reported and some compounds were introduced as the potent inhibitors of PDK2 (16, 17).

Here, in this paper, QSAR studies of a series of N-arylphenyl-2, 2-dichloroacetamide analogues with cytotoxic activity on human non-small cell lung cancer cell line (A 549). which recently designed and synthesized by Li *et al*. (18) have been explored. Among different QSAR models, the best multiple linear regression equation was obtained from GA-PLS models, which was a linear seven-parameter model. Thereafter, a virtual screening study was employed to determine novel biologically active patterns by insertion, deletion and substitution of different substitutes on the primary molecules. The results of this study led to the identification of novel structures, which are potent anticancer agents according to the QSAR model. It also should be mentioned that molecular docking as well as PLIF studies of these compound were also carried out and the promising results were obtained.

## Experimental

*Data set*

The biological data used in this paper are cytotoxic activity of a series of N-arylphenyl-2, 2-dichloroacetamide analogues on human non-small cell lung cancer cell line (A 549). which were designed, synthesized and evaluated for their anticancer activity by Li *et al.* (18). The structural features and biological activity of these compounds are listed in Table 1 The biological data were converted to logarithmic scale (pIC_50_) and then used for subsequent QSAR analysis as dependent variables.

*Molecular descriptors*

The two dimensional structures of the ligands were drawn using ACD chemsketch software. Then the ligands were subjected to minimization procedures by means of an in house TCL script using Hyperchem (Version 8, Hypercube Inc., Gainesville, FL, USA). Each ligand was optimized with different minimization methods such as commonly used molecular mechanics method (MM+) and then quantum based semi-emprical method (AM1) using Hyperchem package. The Z-matrices of the structures were constructed by the software and then transferred to the Gaussian 98 program (19). HyperChem, Gaussian 98 and Dragon softwares (20) were used for calculation of molecular descriptors. Highest occupied molecular orbital (HOMO) and lowest unoccupied molecular orbital (LUMO) energies and molecular dipole moment were calculated by Gaussian98. Quantum chemical indices of hardness (η = 0.5 (HOMO+LUMO)); softness (S = 1 ⁄ η); electronegativity (χ = -0.5 (HOMO-LUMO)); and electrophilicity (ω = χ^2^⁄2η) were calculated according to the equations proposed by Thanikaivelan *et al. *(21). Some chemical parameters including molar volume (V). molecular surface area (SA), hydrophobicity (logP), hydration energy (HE) and molecular polarizability were calculated using Hyperchem software. Dragon calculated different topological, geometrical, charge, empirical and constitutional descriptors for each molecule. 2D autocorrelations, aromaticity indices, atom-centered fragments and functional groups were also calculated by dragon.

In the case of docking procedure, each ligand was optimized with different minimization MM^+ ^then AM1 using HyperChem 8. The output structures were thereafter converted to PDBQT using MGL tools 1.5.6 (22). The three dimensional crystal structure of pyruvate dehydrogenase kinase 2 (PDB ID: 2BU8) was retrieved from protein data bank (23). Co-crystal ligand molecules were excluded from the structures and the PDBs were checked in terms of missing atom types by modeller 9.12 (24). An *in house* application (MODELFACE) was used for generation of python script and running modeller software. Subsequently, the enzymes were converted to PDBQT and gasteiger partial charges were added using MGLTOOLS 1.5.6.

*Model development*

Four different regression methods were conducted for constructing QSAR equations: 1) simple multiple linear regression with stepwise variable selection (MLR) 2) factor analysis as the data preprocessing step for variable selection (FA-MLR), 3) principal component regression analysis (PCRA), and 4) genetic algorithm–partial least squares (GA-PLS). These methods are well substantiated in the QSAR studies, and therefore, these methods are described briefly (25).

Stepwise regression is a semi-automated process of building a model by successively adding or removing variables based solely on the t-statistics of their estimated coefficients. In stepwise regression (26), a multiple-term linear equation was constructed step by step. The basic procedures include (i) recognizing a primary model, (ii) iteratively ‹steppingʹ, that is, repetitively changing the model at the prior step by adding or removing a predictor variable in accordance with the ‹stepping criteriaʹ (in our case, probability of F = 0.05 for inclusion; probability of F = 0.1 for leaving out for the forward selection method), and (iii) terminating the search when stepping is no longer possible given the stepping criteria, or when a known maximum number of steps have been obtained. Particularly, at each step, for determining which one will contribute most to the equation, all variables are reviewed for evaluation (26). The variable will then be applied in the model, and the process starts again. A limitation of the stepwise regression search approach is that it assumes there is a single ‹bestʹ subset of X variables and search for identifying it. There is often no unique ‹bestʹ subset, and whole possible regression models with a similar number of X variables as in the stepwise regression solution should be fitted subsequently to explore whether some other subsets of X variables might be better (27). Here in this study, MLR with stepwise selection and elimination of variables was applied for developing QSAR models using SPSS software (version 21; SPSS Inc., IBM, Chicago, IL, USA). Using MATLAB 2015 software (version 8.5; Math work Inc., Natick, MA, USA), the resulted models were validated by leave-one-out cross-validation procedure to check their prediction ability and robustness.

In FA-MLR method, although classical approach of multiple regression technique was applied as the final statistical tool for developing QSAR relation, factor analysis (FA) (15, 26) was used as the data-preprocessing step to identify the important predictor variables contributing to the response variable and to avoid collinearities among them. In a typical factor analysis procedure, standardizing of the data matrix followed by constructing a correlation matrix is done. An eigenvalue problem is then solved and the factor pattern can be acquired from the corresponding eigenvectors (characteristic vector). The principal objectives of factor analysis (FA) are to display multidimensional data in a space of lower dimensionality with minimum loss of information (explaining >95% of the variance of the data matrix) and to extract the basic features behind the data with ultimate goal of interpretation or prediction. Factor analysis was done on the data set containing biological activity and all descriptor variables, which were to be considered. The factors were extracted by principal component method and then rotated by (VARIMAX) rotation (28).

**Table 1:** Chemical structure of the N-arylphenyl-2, 2-dichloroacetamideanalogues used in this study and their docking binding energy, experimental and cross-validated predicted activity (by GA-PLS) for cytotoxic activity

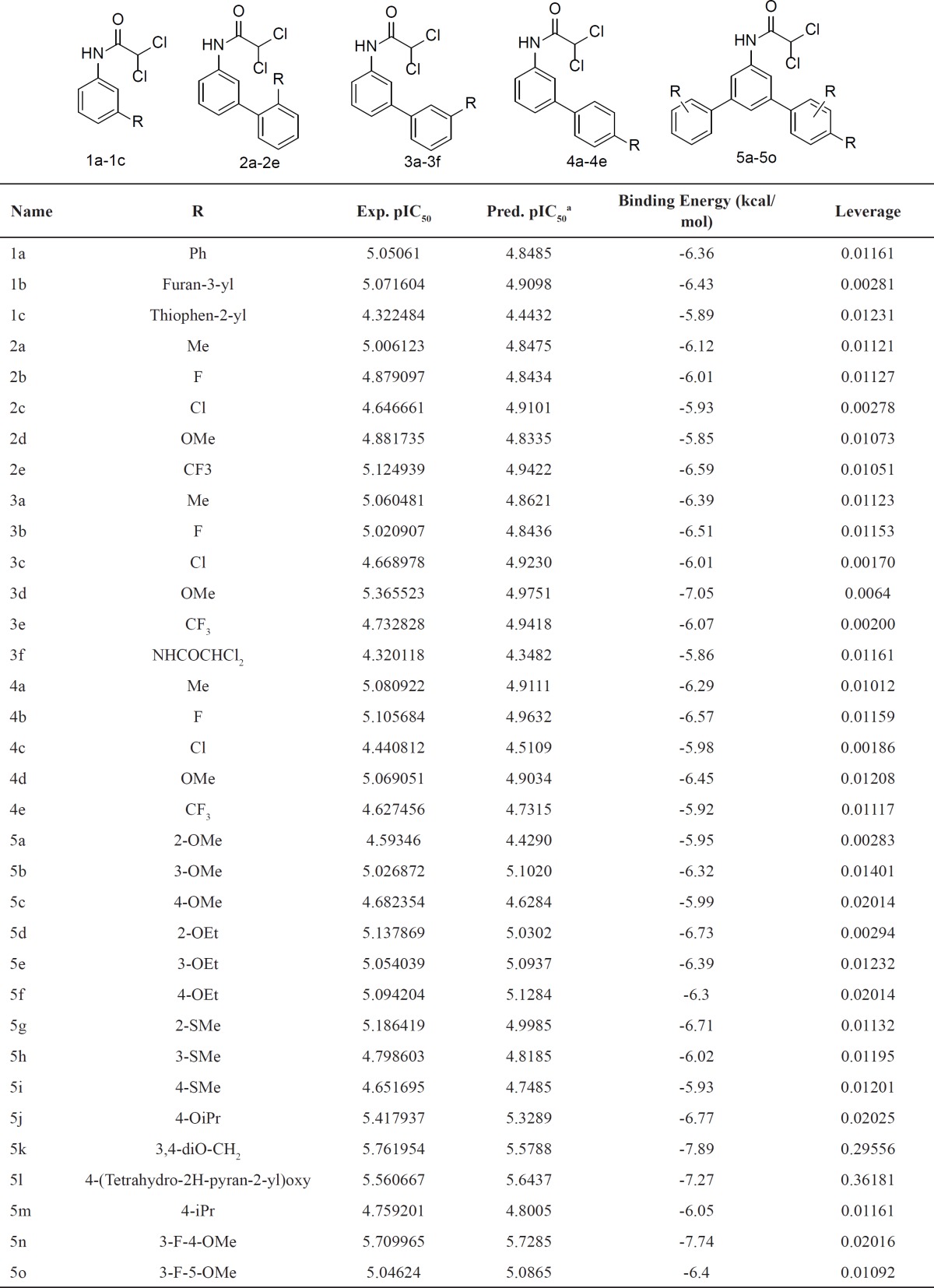

a Cross-validated prediction by

**Table 2:** The results of different QSAR models with different type of dependant variables

**Model**	**Eq.no.**	**MLR Equation**	**n** ^a^	**R** ^2^_c_	**Q** ^2^	**Rmscv**	**Cvcv**	**F**	**SE**	**R** ^2 ^_p_
MLR	**1**	pIC_50_ = 0.010G(O..O) (±0.003) - 0.376nPhX (±0.058) + 0.265DipY(±0.072) -1.574GATS7v (±0.258) + 1.076MATS2e (±0.362)+ 0.205nROR (±0.063) + 0.997MATS7e(±0..401)+7.562 (±0.488)	27	0.917	0.76	0.159	2.78	25.0	0.12	0.70
FA-MLR	**2**	pIC_50_ = 2.152MATS7v(±0.537) + 0.230DipY(±0.083) + 0.244nROR (±0.048) + 0.020Ss (±0.003) +3.538 (±0.204)	27	0.895	0.81	0.197	3.29	19.8	0.22	0.69
PCRA	**3**	pIC_50_ = 0.240 FAC1 (±0.048) + 0.139 FAC2 (±0.048) + 0.114 FAC4 (±0.048) + 0.117 FAC7 (±0.048) + 0.103 FAC9 (±0.048) + 4.969 (±0.047)	27	0.906	0.87	0.168	3.24	19.8	0.27	0.71
GA-PLS	**4**	pIC_50_ = -20.126X3A (±7.555)+3.685MATS7v (±0.391)+ 2.655MATS5p (±0.471) + 0.319DipY(±0.053) + 0.230H-048 (±0.036) -1.084MATS6e (±0.304) -0.637 ASP (±0.234)+8.553 (±1.397)	27	0.943	0.82	0.148	2.99	31.7	0.09	0.87

a Number of molecules of training set used to derive the QSAR modelT

**Table 3:** Correlation coefficient (R^2^) matrix for descriptors represented in multiple linear regression eqn 1.

	**MATS2e**	**MATS7e**	**GATS7v**	**DipY**	**nROR**	**nPhX**	**G(O..O)**
MATS2e	1	-0.160	0.217	0.315	-0.192	-0.207	-0.294
MATS7e		1	0.130	0.013	0.003	-0.048	0.205
GATS7v			1	0.088	0.209	0.090	0.075
DipY				1	-0.233	-0.088	-0.218
nROR					1	-0.134	0.227
nPhX						1	-0.159
G(O..O)							1

**Table 4:** Factor loadings of some significant descriptors after VARIMAX rotation

**Descriptor**	**factor1**	**factor2**	**Factor4**	**Factor7**	**Factor9**	**Communalities**	**PIC50**
pIC50	0.596	0.476	0.389	0.218	-0.169	0.939	
Mp	-0.578	-0.397	-0.210	0.082	-0.049	0.993	-0.021
G(O..O)	0.679	0.445	-0.463	-0.022	-0.023	0.988	0.345
qpos	0.821	0.515	-0.175	0.083	-0.069	0.984	0.234
H-048	0.310	0.371	-0.552	-0.288	-0.128	0.956	0.561
H-052	0.544	-0.019	0.165	0.306	-0.016	0.932	0.508
nROR	0.259	0.364	-0.608	-0.270	-0.067	0.956	0.676
nPhX	-0.159	0.282	-0.063	0.611	0.342	0.970	0.441
X3A	-0.706	-0.386	-0.059	0.198	0.094	0.979	0.398
X3AV	-0.078	-0.757	0.026	-0.125	0.162	0.965	-0.243
lop	-0.482	-0.180	0.415	0.409	-0.243	0.983	-0.129
ATS8p	0.054	-0.527	-0.180	0.387	0.157	0.981	0.256
GATS6m	-0.581	-0.431	-0.133	0.052	0.001	0.940	0.436
GATS8m	-0.590	-0.537	-0.028	0.166	0.122	0.894	0.450
GATS5e	-0.605	0.355	0.037	-0.132	0.079	0.871	0.164
GATS8e	-0.817	-0.100	0.113	0.055	0.027	0.830	0.237
GATS4p	0.120	0.059	0.417	-0.698	-0.151	0.938	0.461
GATS7p	-0.371	0.156	-0.127	-0.118	-0.013	0.903	0.219
GATS4v	0.035	0.086	-0.111	-0.050	0.603	0.965	0.065
MATS5p	-0.145	0.209	-0.616	-0.124	0.086	0.881	-0.432
MATS7v	-0.815	-0.218	-0.018	0.313	0.115	0.944	0.712
MATS4m	-0.288	0.114	-0.008	0.053	-0.008	0.993	0.349
MATS6m	-0.352	-0.088	0.264	0.102	-0.454	0.816	0.415
MATS6e	0.576	-0.346	0.086	-0.040	0.116	0.954	-0.291
MATS7e	0.637	0.047	0.038	-0.098	-0.198	0.858	-0.123
ASP	-0.907	-0.012	-0.017	0.019	-0.072	0.971	-0.293
Ss	0.708	0.586	0.232	0.122	0.032	0.997	0.608
G(N..F)	-0.310	0.721	0.488	-0.046	0.258	0.992	0.341
MAXDP	0.551	0.435	0.145	0.134	.264	0.954	0.326
DipY	-0.027	-0.210	0.357	-0.144	-0.693	0.774	0.632

**Table 5:** Definitions of molecular descriptors present in the models

**No.**	**Descriptors**	**Brief description**
1	ATS8p	Broto-Moreau autocorrelation of a topological structure - lag 8 / weighted by atomic polarizabilities
2	MATS7v	Moran autocorrelation - lag 7 / weighted by atomic van der Waals volumes
3	MATS4m	Moran autocorrelation - lag 4 / weighted by atomic masses
4	MATS6m	Moran autocorrelation - lag 6 / weighted by atomic masses
5	MATS5p	Moran autocorrelation - lag 5 / weighted by atomic polarizabilities
6	MATS6e	Moran autocorrelation - lag 6 / weighted by atomic Sanderson electronegativities
7	MATS7e	Moran autocorrelation - lag 7 / weighted by atomic Sanderson electronegativities
8	GATS4v	Geary autocorrelation - lag 4 / weighted by atomic van der Waals volumes
9	GATS7v	Geary autocorrelation - lag 7 / weighted by atomic van der Waals volumes
10	GATS6m	Geary autocorrelation - lag 6 / weighted by atomic masses
11	GATS4p	Geary autocorrelation - lag 4 / weighted by atomic polarizabilities
12	GATS7p	Geary autocorrelation - lag 7 / weighted by atomic polarizabilities
13	GATS8e	Moran autocorrelation - lag 8 / weighted by atomic Sanderson electronegativities
14	X3A	average connectivity index chi-3
15	X3AV	average valence connectivity index chi-3
16	H-048	H attached to C2(sp3) / C1(sp2) / C0(sp)
17	H-052	H attached to C0(sp3) with 1X attached to next C
18	G(O..O)	sum of geometrical distances between O..O
19	Lop	Lopping centric index
20	nPhX	number of X-C on aromatic ring
21	nROR	number of ethers (aliphatic)
22	ASP	Asphericity
23	DMY(DipY)	Molecular dipole moment at Y-direction

**Table 6:** Structural modification of N-arylphenyl-2, 2-dichloroacetamide analogues and their predicted activities and docking binding energy

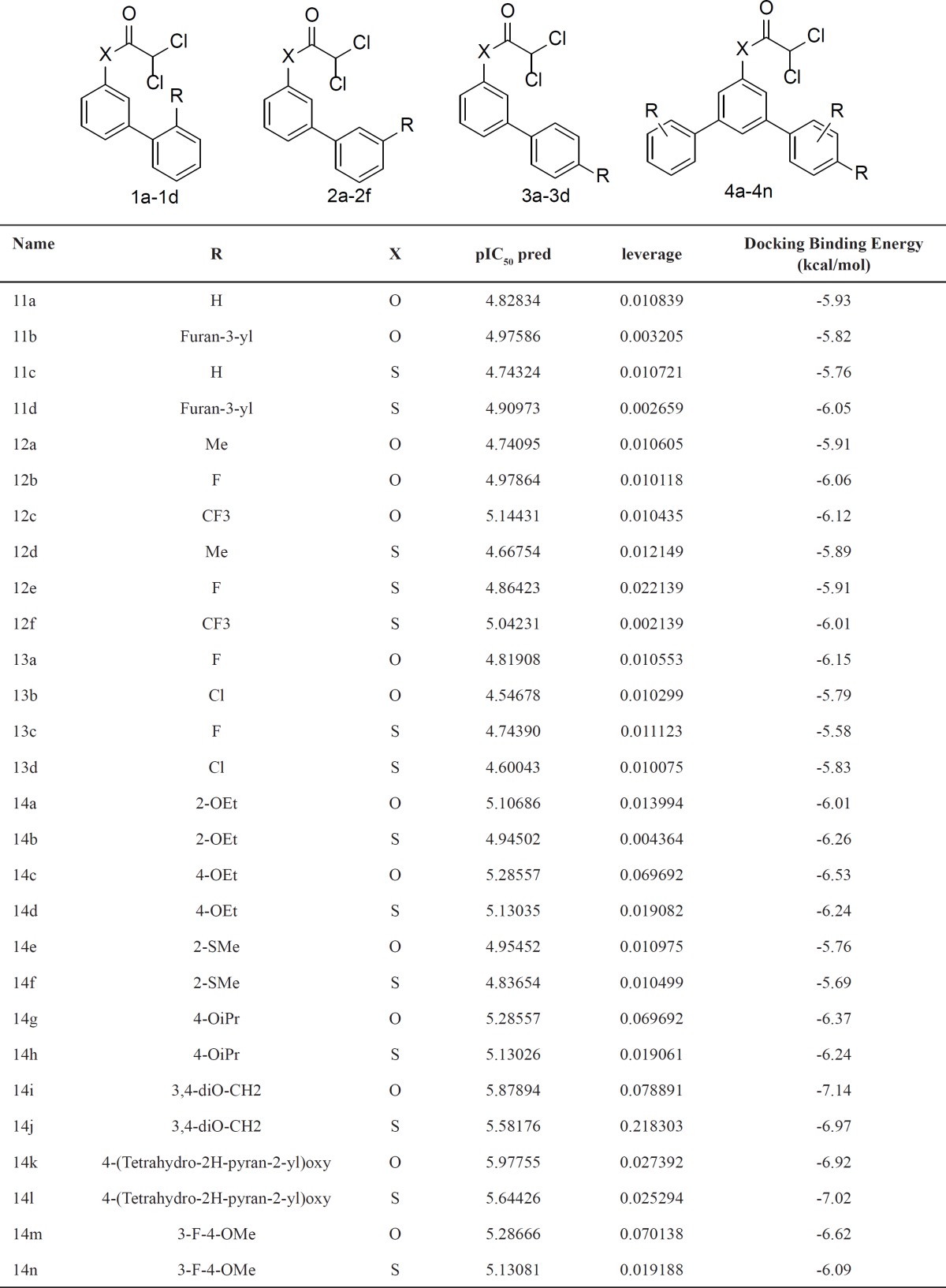

**Figure 1:**
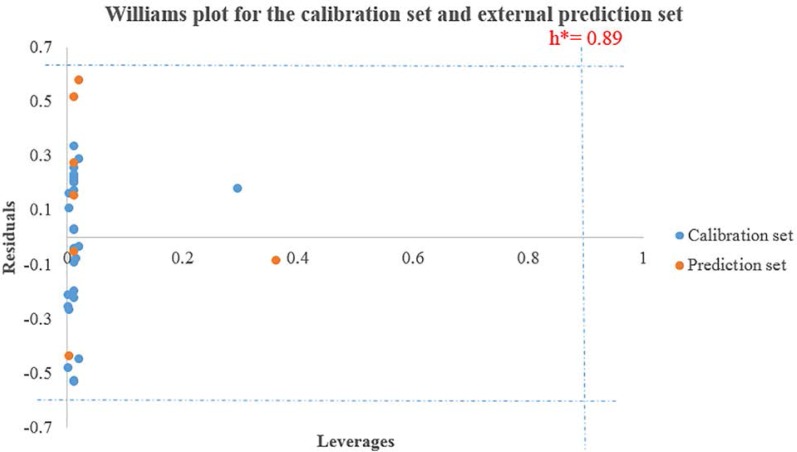
Williams plot for the training set and external prediction set for cytotoxic activity of N-arylphenyl-2,2-dichloroacetamide analogues

**Figure 2:**
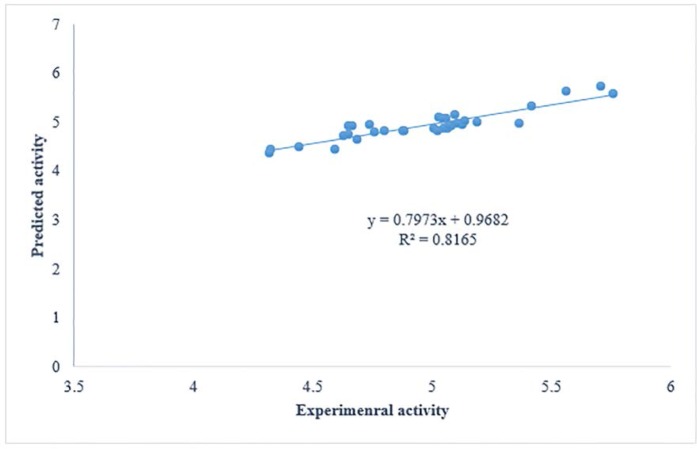
Plots of cross-validated predicted values of activity by GA-PLS against the experimental values

**Figure 3.**
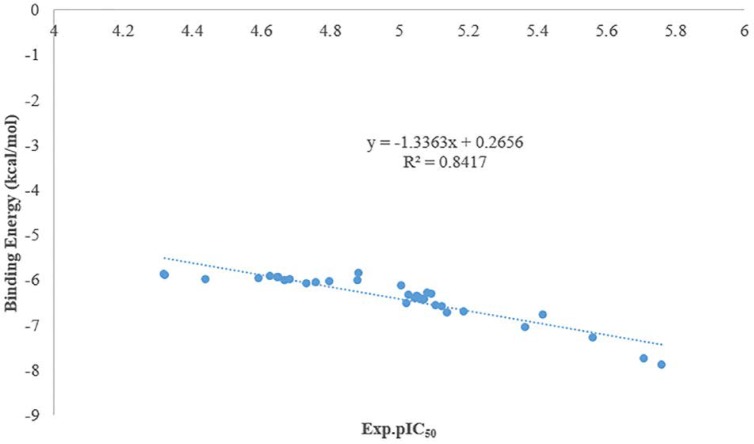
Plots of experimental pIC_50_ values versus docking binding energy

**Figure 4:**
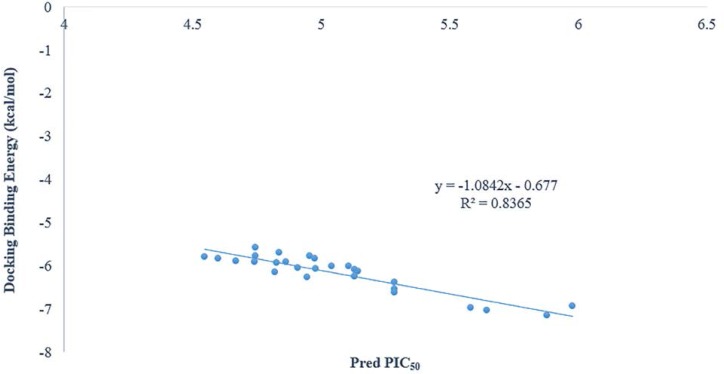
Plots of predicted pIC_50_ values versus docking binding energy

**Figure 5:**
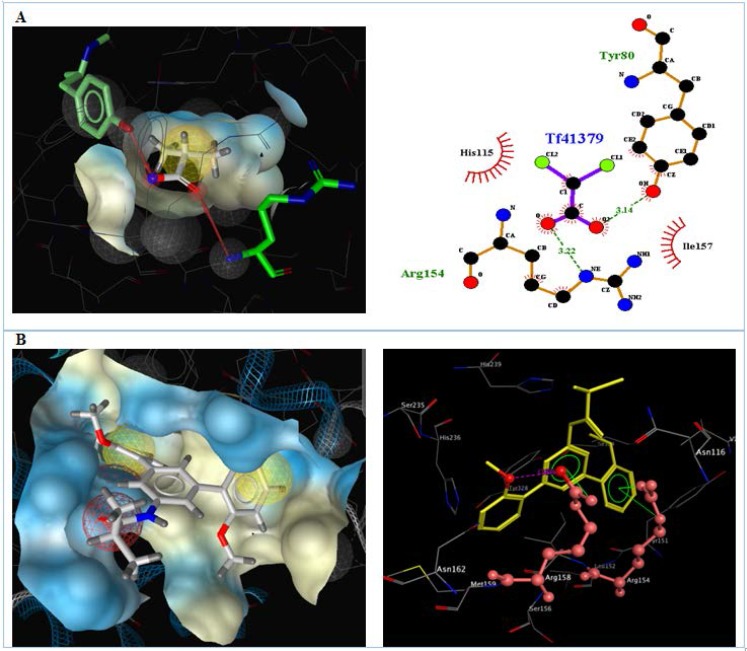
Interactions of A) DCA and B) compound 4d with the residues in the binding site of PDK (2BU8) receptor.

**Figure 6:**
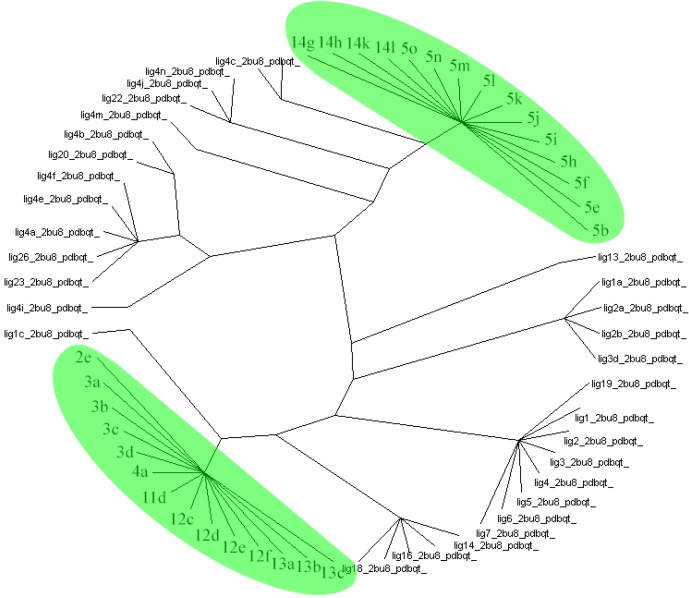
AuposSOM results for poses of docking.

Along with FA-MLR, PCRA was also tried for the data set. In this method (15, 26), factor scores that obtained from FA are used as the predictor variables. PCRA has a benefit that collinearities among X variables are not a disturbing factor and that the number of variables included in the analysis may exceed the number of observations (29). While the main purpose of FA-MLR is to identify relevant descriptors, in PCRA model all descriptors are supposed to be important.

Genetic algorithms (GA) generate solutions to optimization problems using techniques inspired by natural evolution, such as inheritance, mutation, selection, and crossover.

Partial least square (PLS) is a generalization of regression, that can handle data with forcefully correlated and numerous X variables (30). It gives reduced solution, which is statistically more robust and reliable than MLR. The linear PLS model finds ‹new variablesʹ (latent variables or X scores) that are linear combination of the original variables. To avoid overfitting, a strict test for the significance of each consecutive PLS component is necessary and then stopping when the components are non-significant. Cross-validation is a practical and credible method of testing this significance (31). Application of PLS thus allows the construction of larger QSAR equations while still avoiding over fitting and eliminating most variables. Usually PLS is applied in combination with cross-validation to obtain the optimum number of components (26, 32, 33). In the GA-PLS procedure, in addition to the best set of descriptor, the optimum number of concealed variable must be determined. Here, for each subset of descriptors (i.e., for each chromosome of the GA), a PLS model was developed separately and therefore the number of latent variables was optimized. The PLS regression method was applied the NIPALS-based algorithm existed in the chemometrics toolbox of MATLAB software. Leave-one-out cross-validation procedure was used to obtain the optimum number of factors based on the Haaland and Thomas F-ration criterion (26, 34). The MATLAB PLS toolbox developed by eigenvector company was used for PLS and GA modeling. All calculations were run on a core i7 personal computer (CPU at 6 MB) with Windows 7 operating system.

*Model validation*

Statistical parameters including correlation coefficient (R^2^), standard error of regression (SE), and variance ratio (F) at specified degrees of freedom were used for validating the goodness-of-fit of the resulted QSAR models. The generated QSAR equations were also validated by leave-one-out cross-validation correlation coefficient (Q^2^), root mean square error of cross-validation (RMScv) and cross validation cross validation (Cvcv). According to Tropsha *et al.* (35) the predictive ability of a QSAR model should be tested on an external set of data that has not been taken into account during the process of developing the model. Therefore, as it was shown in table 1, an external test set composed of randomly selected 7 molecules (for example 1a, 2a, 2e, 3c, 4d, 5 h. and 5m) were applied to determine the overall prediction ability of the resulted models. It should be emphasized that we carried out each QSAR model with more than 3 test set and the best equation was considered as the best model.

*Applicability domain*

One of the great uses of a QSAR model is based on its precise prediction ability for new compounds. A model validation is just within its training domain, and new compounds must be appraised as belonging to the domain before the model is applied. The applicability domain is appraised by the leverage values for each compound. A Williams’s plot (the plot of standardized residuals versus leverage values (h)) can then be used for an immediate and simple graphical detection of both the response outliers (Y outliers) and structurally influential chemicals (X outliers) in our model. In this graph, the applicability domain is established inside a squared area within ±x (standard deviations) and a leverage threshold h*. The threshold h* is generally fixed at 3(k + 1) ⁄ n (k is the number of model parameters and n is the number of training set compounds), whereas x = 2 or 3. Prediction must be considered unreliable for compounds with a high leverage value (h > h*). From the other point of view, when the leverage value of a compound is lower than the threshold value, the probability of agreement between observed and predicted values is as high as that for the training set compounds (36, 37).

*Docking procedure*

The docking simulations were carried out by means of an *in house *batch script (DOCKFACE) for automatic running of AutoDock 4.2 (38) in a parallel mode using all system resources. In all experiments Genetic algoritm search method was used to find the best pose of each ligand in the active site of the target enzyme. Random orientations of the conformations were generated after translating the center of the ligand to a specified position within the receptor active site, and making a series of rotamers. This process was recursively repeated until the desired number of low-energy orientations was obtained. No attempt was made to minimize the ligand-receptor complex (rigid docking). For Lamarckian GA method; 27,000 maximum generations; 2500000 maximum No. of evaluations, 150population size, mutation rate of 0.02; and a crossover rate of 0.8 were used. A grid box of 50×50×50 points in x, y, and z direction with a grid spacing of 0.375 Å was built. No. of points in x, y and z was 50, 40 and 81 respectively. 

*Protein ligand interaction fingerprint (PLIF)*

In order to perform PLIF studies on docking results, the poses of docking were extracted from dlg files using an *in house* vb.net application (pre Aupos SOM) (39). The resulted pdbqts and the receptor were converted to mol2 using Open Babel 2.3.1. The resulted mol2 files were submitted to Aupos SOM 2.1 web server (40-42). Two training phases with 1000 iterations were set in the self-organizing map settings of Aupos SOM conf files. Other parameters of the software were remained as default. The output files were subjected to Dendroscope 3.2.10 for visualization of the results (43, 44). The PLIF parameters were set as default of the AuPos SOM v2.1 Web Application.

## Results and Discussion

In this study, we executed a detailed QSAR study using a combination of chemical, electronic and substituent constant, to explore structural parameters affecting cytotoxic activity of novel N-arylphenyl-2, 2-dichloroacetamide analogues. Among the different chemometrics tools available for modeling the relationship between the biological activity and molecular descriptors, four methods (i.e. stepwise MLR, FA-MLR, PCRA, and GA-PLS) were applied and compared here. A comparison between stepwise FA-MLR and MLR will indicate which variable selection method (stepwise or FA) is well suited for MLR, whereas a comparison between FA-MLR and PCRA reveals for modeling of the studied biological activities, using original descriptors selected based on factor loading or using the factor scores calculated based on all calculated descriptors results in more suitable model. Eventually, GA-PLS, which is assumed to produce the most useful model, was employed, and its results were compared with the other employed models. 

*MLR modeling*

Firstly, separate stepwise selection-based MLR analyses were performed using different types of descriptors, and then, a MLR equation was obtained utilizing the pool of all calculated descriptors. As it was shown in Table 2, statistical parameters such as correlation coefficient (R^2^). correlation coefficient (R^2^_p_) of test set, standard error of regression (SE), and variance ratio (F) at specified degrees of freedom, leave-one-out cross-validation correlation coefficient (Q^2^), cross validation cross validation (Cvcv) and root mean square error of cross-validation (RMScv) were used for validating the goodness-of-fit of the resulted QSAR equations. Equation 1 was selected as the best equation in the MLR model. The selected variables demonstrate that quantum (DipY), geometrical (G (O..O)), 2D autocorrelations (MATS2e, MATS7e, GATS7v), and functional (nPhX, nROR) descriptors affect the cytotoxic activity of the studied compounds.

A small difference between the conventional and cross-validate correlation coefficients of the different MLR equations reveals that none of the models are over fitted, which can be partially attributed to absence of collinearity between the variables in one hand and using of no extra variables in the other hand. The correlation coefficient (r^2^) matrix for the descriptors used in MLR equation 1, shows that no significant correlation exists between pairs of descriptors (Table 3).

*FA-MLR and PCRA*

It was discovered that five factors could explain the data matrix to the extent of 96.3%, from the factor analysis on the data matrix consisting of the pIC50 and calculated molecular descriptors. Table 4 shows that the biological activity is highly loaded with factors 2 and especially 1. The highest loading values for factor 2 are associated with X3AV, and G (N.F) descriptors whereas Ss, ASP, qpos, G(O.O), MATS7v, MATS7e, GATS5e and GATS8earethe highly loaded descriptors of factor 1. Table 4 revealed that, factors 1 and 2 are moderately loaded with cytotoxicity activity. Interestingly, the former possessed the highest loadings from geometrical (G(O..O), ASP), constitutional (Ss), 2D autocorrelations (MATS7v, MATS7e, GATS5e, GATS8e) and charge (qpos) descriptors whereas the latter is containing the information from topological (X3Av) and geometrical (G (N.F)) descriptors. As it was shown in equation 6, the highly loaded descriptors of factors 1, 2, 4, 7and 9 (instead of applying the pool of all calculated descriptors) can be considered as the source of molecular descriptors for QSAR model building. So, the probability of obtaining chance models is decreased (45).

The subsequent FA-MLR equation using highly loaded descriptors is shown in Table 2, Eq.2.

*PCRA*

When factor scores were used as the predictor parameters in a multiple regression equation (instead of their highly loaded descriptors), a predictive QSAR model with factor scores number 1, 2, 4, 7and 9 as input variable was obtained (Eq. 3). This equation shows statistical quantities similar to those obtained by FA-MLR method (Table 2). However, it shows slightly higher calibration and lower cross-validation statistics with respect to Eq 2. This shows a sign of overfitting since the factors considered in Eq. 3 have information from irrelevant descriptors too. Considering this information in modeling may apparently increase the model variances (i.e., R^2^) but they are not useful for prediction. On the other hand, the advantage of the QSAR model obtained by PCRA is that the factors appeared in the MLR equation 3 are orthogonal. The regression coefficients calculated for such variables are more stable and thus are closer to the real values. In addition, from the factor scores used, significance of the original variables for modeling the activity can be obtained. Factor score 1 indicates the importance of geometrical (G(O..O), ASP). constitutional (Ss), 2D autocorrelations (MATS7v, MATS7e, GATS5e, GATS8e) and charge (qpos) descriptors. The factor score 2 indicates importance of topological (X3Av) and geometrical (G (N.F)) descriptors, and factor score 4 signifies the importance of functional (nROR) and 2D autocorrelations (MATS5p) descriptors. The factor score 7reveals the importance of the 2D autocorrelations parameters (GATS4p) and functional (nPhX) descriptors. The factor score 9 signifies the importance of quantum (DipY) and 2D autocorrelations (GATS4v) descriptors. 

*GA-PLS*

In PLS analysis, having decomposed the descriptors data matrix to orthogonal matrices, then the scores are constrained to have inner relationship with the dependent variables. Hence similar to PCRA, the multicollinearity problem in the descriptors is omitted by PLS analysis. Genetic algorithm was applied to find the more useful set of descriptors in PLS modeling. So, many different GA-PLS runs were done using different initial set of populations. The results of this model are summarized in Table 2.

As it is shown in Table 2 Eq 4, a combination of quantum (DipY). 2D autocorrelations (MATS7v, MATS5p, MATS6e), atom- centered fragments (H-048), geometrical (ASP) and topological (X3A) descriptors have been selected by GA-PLS to account for the cytotoxic activity of N-arylphenyl-2, 2-dichloroacetamide analogues. The resulted GA-PLS model possessed very high statistical quality parameters (i.e., R^2^ = 0.94and Q^2^ = 0.82). The predictive ability of the model was measured by application to 7 external test set molecules. The squared correlation coefficient for prediction was 0.87, and standard error of prediction was 0.099.

Table 2 shows that none of the proposed QSAR models were obtained by chance and the GA-PLS model because of its greatest statistical parameters is the best predictive model.

The brief description of the descriptors used by QSAR models are summarized in Table 5.

*In silico screening*

*In silico *research in medicine is thought to have the potential to speed the rate of discovery, predicting and identifying new biologically active compounds while reducing the need for expensive lab work and clinical trials. One way to attaint his is by generating and screening drug candidates more effectively. On the other hand, the *in silico* procedure minimizes the time and cost associated with identifying new leads (46, 47). 

A virtual screening was applied by deletion, insertion and substitution of different substitutes on the parent molecules and the effects of the structural modifications on the biological activity were investigated. Then, the domain application of QSAR model was determined to apply the model for screening new compounds. The applicability domain (AD) of QSAR model was used to verify the prediction reliability, to identify the troublesome compounds and to predict the compounds with accep table activity that falls within this domain.

The important descriptors selected by GA-PLS model (because of its greatest statistical parameters compared to the others it was chosen as the best model) could be used for designing new active compounds. Analyzing the model applicability domain (AD) in the Williams plot (Figure 1) of the GA-PLS model based on the whole data set, appeared that none of the compounds were identified as an obvious outlier for the cytotoxic activity if the limit of normal values for the Y outliers (response outliers) was set as 2.5 times of the standard deviation units. As it is cleared, none of the compounds have leverage (h) values greater than the threshold leverages (h*). The warning leverage (h*), was found to be 0.89 for the developed QSAR model. The compounds that had a standardized residual more than three times of the standard deviation units were considered to be outliers. For both the training set and prediction set, the presented model matches the high quality parameters with good fitting power and the capability of assessing external data. Moreover, almost all of the compounds were within the applicability domain of the proposed model and were evaluated accurately. While chemicals with a leverage value higher than h* were considered to be influential or high leverage chemicals (26, 34).

Next, the *in silico *screening was used to the design of new compounds with potential cytotoxic activity according to the developed QSAR model and was validated by the developed GA-PLS model. So, the compounds in Tables 1 with IC_50_ <9.0μm were selected as template due to their good cytotoxic activity. Then, the *in silico* screen was applied by substituting different bioisosteric groups (O, S) in the place of-NH group; the results of this investigation are summarized in Table 6.

The model tolerated various bioisosteric changes of NH groups by sulfur and oxygen groups. Since all of the studied derivatives were within the applicability domain. Among different designated molecules, the compound 4c, 4g, 4i, 4j, 4k, 4m showed the best activity (pIC50 >5.25). Thus, in order to clarify the relation between the activities of the compounds with different functional groups, this compound was chosen for more structural modification. As it was shown in **table 9**, some esteric and thioesteric derivatives of this class of anticancer compounds have a good potentially for becoming anticancer agent. Finally, this result confirms the reliability of the models and it shows that with the aim of the QSAR model and use of *in silico* screening, it is possible to identify new synthetic compounds for drug discovery.

The proposed QSAR models have all conditions to be considered as predictive models. Firstly, all have correlation coefficient of cross-validation (Q^2^) larger than 0.5 and of prediction (r^2^) higher than 0.6. Thus, according to great statistics, GA-PLS can be considered as the most predictive model. According to the cross-validation results all models have Q^2^> 0.7 and can be considered predictive models. To have a consideration on the cross-validated prediction results, the predicted activity data are plotted against the experimental activities in Figure 2. As it was mentioned in the article, the least scattering of data was obtained from GA-PLS.

*Docking Studies*

Docking is frequently used to predict the binding orientation of small molecule drug candidates to their protein targets in order to in turn predict the affinity and activity of the small molecule. Hence docking plays a great role in the rational design of drugs. DCA stimulates the activity of the enzyme PDH through inhibition of the enzyme PDKs. The crystal structure of PDK2 in complex with DCA has been acquired, and it shows that DCA indwells the pyruvate binding site in the N-terminal regulatory (R) domain (1).

Here, in this study docking studies were carried out on the compounds in Table 1 and 6 to find their binding site, binding modes and the best direction on the base of their binding energy. The docking simulations were carried out by means of an *in house *batch script (DOCKFACE) for automatic running of AutoDock 4.2 in a parallel mode using all system resources. Having completed the docking process, then the protein–ligand complex was analyzed to investigate the type of interactions. Top ranked binding energies (kcal/moL) in AutoDock dlg output file were considered as response in each run. Docking results were supported almost by high cluster populations. The conformation with the lowest binding energy was considered as the best docking result in each case.

As it was shown in figure 3 there is a good relationship between experimental pIC_50_ and docking binding energy. Hence, our docking protocol can discriminate between the ligand (active) and decoys (non-active). The validated docking procedure was also applied to our designed ligands. Figure 4, shows that this correlation was also existed between predicted pIC_50_ of QSAR studies and docking binding energy. Compounds 14i-m based on the best docking binding energy can be considered as good candidates for synthesis.

The results for each ligand were compared to its corresponding co-crystal ligand. Hydrogen bindings between docked potent agents such as 3g and the PDK receptor (2BU8) was analyzed using Autodock tools program (ADT, Version 1.5.6). ligplotv.4.5.3 (48) and Ligand Scout 3.12 (49). As it was shown in figure 5, a hydrogen bond acceptor interaction exists between oxygens of carboxyl group of co-crystal ligand (DCA) and Arg 154, Tyr 80 in receptor (figure 5A). Meanwhile, a hydrogen bond acceptor interaction existed between oxygen of methoxy group of 4d with Arg158, in receptor. There is also exists an arene-cation interaction between the phenyl group that bearing amide substituent with Arg158 and an arene-cation interaction between the phenyl group that bearing methoxy group in the receptor with Arg154 (Figure 5B).

Protein ligand interaction fingerprint (PLIF) studies could be used as a more reliable analysis technique (40). This method makes it possible to study the effect of different starting states of the structures on generated poses as well as their corresponding vector of contacts towards receptor during docking procedure. For this purpose, the docking of all 34 compounds of QSAR study as well as our designated compounds were carried out, then all generated poses of the ligands were subjected to Aupos SOM 2.1 to calculate their contact vectors within the receptor binding cavity. In this procedure, the contacts between the structures and the protein comprise of hydrophobic, hydrogen bonding and coulombic interactions. The resulted vectors of contacts are then analyzed using self-organizing map as implemented in Aupos SOM software. The output of self-organizing map is a classification pattern for ligands. For visualization of the results, the output files were subjected to Dendroscope 3.2.10. To the best of our knowledge, ligands in the same subgroup may show a similar behavior. As it was shown in figure 6, designated ligands such as 14g, 14h, 14k and 14l are clustered in the 5b (the best compound due to its greatest IC_50_), 5e, 5f and 5i-o subgroup. Meanwhile, compounds 2e, 3a-d, 11d, 12c-f, 13a-c are clustered in the same subgroup. So these compounds may have a similar behavior as theirs and can be good candidates for synthesis.

## Conclusion

In this study, four different QSAR modeling methods, MLR, FA-MLR, PCR and GA-PLS as well as FWA were used in the construction of a QSAR model for cytotoxic activity of N-arylphenyl-2, 2-dichloroacetamide analogues and the resulting models were compared. As it was shown in the article, having performed GA before the calibration, a regression model with enhanced predictive power would be obtained. The reliability, accuracy and predictability of the proposed models were evaluated by various criteria, including cross-validation, the root mean square error of prediction (RMSEP), root mean square error of cross-validation (RMSECV), validation through and Y-randomization. It was also shown that the proposed model is a useful aid for reduction of the time and cost of synthesis and biological evaluation of DCA analogues. Moreover, the results confirm that among the applied models, the GA-PLS is superior for prediction of the pIC50 of DCA analogues. The statistical parameters of the four different chemometrics methods used in this study are represented in Table 2. Some models represent high goodness of fit (measured by R^2^), whereas that obtained by GA-PLS is significantly better than that of the other models. To the best of our knowledge, GA-PLS is the best choice for the prediction purpose of QSAR study, and for descriptive purpose it should be better to use MLR method. The cross-validation statistics reported in Table 2 suggest the higher prediction ability of the GA-PLS model. This can be ascribed to the exploit of a large number of descriptors by GA-PLS in compared to the MLR. The study suggests the importance of dipole moment in y-direction (DMY), 2D autocorrelations and a sphericity (ASP) of molecules for DCA analogues cytotoxic activity. It is clearly understood that 2D autocorrelation descriptors such as MATS7v, MATS6e, MATS5p, geometrical descriptors such as ASP, atom- centered fragments likeH-048, topological descriptors like X3A and quantum chemical parameter (DMY) are important structural parameters that significantly influence the cytotoxic activity. The 2D autocorrelation descriptors depict the topological structure of the compounds, but are more complicated in nature with respect to the classical topological descriptors. The calculation of these descriptors includes the summations of different autocorrelation functions corresponding to different structural lags and leads to different autocorrelation vectors corresponding to the lengths of the substructural fragments. As a result, these descriptors address the topology of the structure or parts thereof in association with a specific physicochemical property. According to the developed QSAR model, *in silico *screening was applied and new compounds such as 4c, 4g, 4i, 4j, 4k, and 4m with potential cytotoxic activity were suggested for synthesis.

There was a good correlation between docking binding energy and experimental pIC_50_. The molecular docking study revealed that there is an arene-arene interaction between phenyl group the phenyl group that bearing amide substituent with Arg158 and an arene-cation interaction between the phenyl group that bearing substituents with Arg154 in the receptor. As it was shown in figure 4, based on the substituent on phenyl group, a hydrogen bond acceptor interaction also existed with the substituent and Arg158 in receptor. The docking results were also subjected to PLIF studies and compounds 11d, 12c-f, 13a-c, 14g, 14h, 14k and 14l are introduced as a good candidates for synthesis. 
